# Addition of Vital Wheat Gluten to Enhance the Quality Characteristics of Frozen Dough Products

**DOI:** 10.3390/foods5010006

**Published:** 2016-01-06

**Authors:** Virginia Giannou, Constantina Tzia

**Affiliations:** Laboratory of Food Chemistry and Technology, School of Chemical Engineering, National Technical University of Athens, 5 Iroon Polytechniou St., Polytechnioupoli Zografou, 15780 Athens, Greece; vgiannou@chemeng.ntua.gr

**Keywords:** frozen dough, gluten, quality, bread

## Abstract

The aim of this study was to enhance the quality and sensory characteristics of bread made from frozen dough. Both white and whole-wheat flour were used. In order to improve dough strength and stability during frozen storage, samples were supplemented with vital wheat gluten at the levels of 2%, 4%, 5%, and 6% of flour weight. The characteristics of baked samples were determined through weight loss, specific volume, crust, and crumb color, texture, and sensory evaluation. Dough behavior at sub-zero temperatures was further examined for control samples and samples with 6% gluten using Differential Scanning Calorimetry (DSC), while their low molecular sugar content (fructose, glucose, sucrose) was measured using High Pressure Liquid Chromatography (HPLC), as it can be associated with yeast viability and dough freezing point depression. The most stable samples were those with 4% and 6% gluten (for white flour) and those with 4% and 5% gluten (for whole-wheat flour). Gluten addition raised the freezing point of dough samples and preserved low molecular sugar generation after prolonged storage.

## 1. Introduction

Storage at low-temperatures is a preservation method widely applicable in the food industry as it temporarily retards the physicochemical or biological processes which result in the quality degradation of foods. Bakery products in particular, when stored at freezing conditions, can remain stable for weeks or months [1]. Besides the elongation of the shelf-life, freezing of bakery products also facilitates their handling, trading, and retail ability [2,3].

However, prolonged frozen storage inevitably impairs the quality characteristics of the dough [4]. As a result, yeast’s viability and gas production ability decrease and gluten network’s stability and CO_2_ retainability decline [5,6].

In order to minimize quality loss and preserve dough properties during freezing, the adjustment of processing conditions or the use of different additives/ingredients have been suggested [7]. In the current study, the incorporation of vital wheat gluten in bread samples prepared from frozen dough was studied. This was impelled by the fact that vital wheat gluten is reported to increase dough and bread yields and improve mixing tolerance and bread crumb texture while additionally enhancing the protein level and hence the nutritional value of the product [8,9,10]. Besides, wheat is unique among other grains due to its proteins (mainly gliadins and glutenins), which when hydrated, are disrupted and transformed into gluten. This results in the generation of a cohesive dough network able to trap and retain the gases formed during breadmaking, (mixing, fermentation, baking) and expand [11,12,13]. Therefore, gluten is considered essential for providing bakery products with superior quality characteristics [14,15].

The objective of this research was to study the effect of the addition of vital wheat gluten, at different levels, on the physical and sensory characteristics of frozen bread dough products, during storage.

## 2. Materials and Methods

### 2.1. Raw Materials

The raw materials used for dough preparation were: strong white wheat flour (gluten content: 14%) and whole grain wheat flour (gluten content: 13%) from “Sarantopoulos Flourmill” (Keratsini, Greece), ascorbic acid (food grade—Merck, Germany) and commercially available sugar, salt, dry yeast, and vegetable shortening. Vital wheat gluten was obtained from “Roquette Italia S.p.A.” (Cassano Spinola, Alessandria) and had the following specifications: protein content (dry basis) < 83%, starch (approx.) 10%, fats (approx.) 3%, cellulose content (approx.) 0.5%, water retention (approx.) 160%, drying loss > 8%, particle size (residue on 200 μm) > 1%.

### 2.2. Dough Preparation

Raw materials were weighed in the following proportions: 500 g of flour, 300 g of water (60% *w*/*w*—flour basis), 10 g of dry yeast (2% *w*/*w*), 20 g of sugar (4% *w*/*w*), 15 g of vegetable shortening (3% *w*/*w*), 10 g of salt (2% *w*/*w*), and 0.05 g of ascorbic acid (100 ppm). Vital wheat gluten was added at the levels of 2%, 4%, 5%, and 6% of flour weight respectively (without weight adjustment of the other ingredients). Control samples (without vital wheat gluten) were also prepared for comparison. In each batch, yeast was pre-hydrated with the water and all dough ingredients were placed in a “Kenwood” domestic blender (Kenwood Chef KM400, Kenwood, UK) and mixed for 2 min at a low speed (speed 2) and for 8 min at a medium speed (speed 4). As soon as dough was formed, it was separated in samples of 80 g, which were slightly round shaped by hands. Samples were placed in aluminum pans as soon as possible, wrapped with plastic membrane, weighed, and placed in the freezer (Iberna SCO 50, Iberna, Italy) at −20 ± 2 °C. The same procedure was followed both for samples prepared from white and whole-wheat flour.

### 2.3. Breadmaking

Samples remained under frozen storage for almost four months (111 days for samples from white flour and 110 days for those from whole-wheat flour). In each sampling (every two weeks on average) three samples were withdrawn from the freezer and placed in an incubation chamber (Bekso EB1N, Bekso, Brussels, Belgium) at 25 °C for 195 min in order to thaw (165 min) and proof (30 min). Soon after thawing was completed, dough texture characteristics were counted using one of the samples, while the remaining were allowed to proof and baked into a laboratory oven with air circulation at a temperature of 180°C, for 35 min. After baking, bread samples were left to cool down for about 30 min at ambient temperature and were subjected to the following measurements and analysis.

### 2.4. Specific Volume

The specific volume of bread samples was determined as the volume/weight ratio by weighing baked samples and measuring their volume using the AACC 10-05.01 rapeseeds displacement method [16].

### 2.5. Color Measurement

Crust and crumb color of baked samples was measured using a Minolta CR/200 chromatometer (Minolta Company, Chuo-ku, Osaka, Japan), which displays the L*, a*, b*, color parameters for every sample according to the CIELAB system of color measurement [17]. Color variation (chroma) was estimated according to the following equation:

$$C*=\sqrt{a{*}^{2}+b{*}^{2}}$$

### 2.6. Texture Analysis

Dough and bread firmness was determined using a TA-XT2i (Stable Micro Systems Ltd., Godalming, Surrey, UK) Texture Analyzer. Dough samples, directly after thawing/proofing, as described above, were subjected to a two-cycle compression test using the SMS P/45C cone probe (test speed 3 mm/s, distance 15 mm).

Whole/uncut bread samples were subjected to crust texture analysis and thereafter carved in the middle (vertically), using a double slice knife of 1 cm thickness. The resulting slices (three parts) were used for crumb firmness measurement. In both cases (crust and crumb), a cut test was applied using the TA-45 craft knife (test speed 3 mm/s, distance 15 mm).

All the above measurements/analyses were performed in triplicate.

### 2.7. Sensory Evaluation

Samples of baked bread were assessed for their volume, crust and crumb color, fissures, crust firmness, and crumb elasticity using Quantitative Descriptive Profile Analysis (QDP). A panel of five assessors, trained in sensory evaluation principles and in baked products in particular, was used. Assessors were guided to evaluate samples with a scale scoring from 0 to 6 (in increasing intensity of each characteristic). Two replicates of each sample were performed and results were presented in cobweb diagrams [8].

### 2.8. DSC Studies

The thermal behavior of certain samples (control and 6% gluten made from white flour) was monitored using a Perkin Elmer DSC 6 (PerkinElmer Inc., Wellesley, MA, USA) instrument with Pyris Manager Thermal Analysis Software. The DSC instrument was calibrated using indium as a standard (Tm = 156.6 °C, ΔHm = 28.5 J/g). Samples (~30 mg) were placed into Perkin Elmer aluminum DSC pans of 50 μL and hermetically sealed. An empty sealed pan was used as a reference sample. Liquid nitrogen was used as a coolant at a rate of 10°C/min while nitrogen gas (99.9% purity) was used to minimize water condensation in the measuring cell. The temperature range used for DSC analysis of dough samples was from −50 to 50°C. The samples used for DSC analysis, prepared as described above, were frozen, stored overnight, and thawed according to the standard procedure.

### 2.9. Low Molecular Sugar Content

#### 2.9.1. Sample Preparation

Dough samples (30 g) with 6% gluten as well as control samples, directly after thawing/proofing as described above, were brought into a volumetric flask of 100 mL and 5 mL of potassium ferrocyanide [Κ_4_[Fe(CN)_6_]·3H_2_O] (Riedel-de Haen AG, Germany) solution (dissolution of 10.6 g of salt in water with final volume of 100 mL) and zinc acetate [Zn(CH_3_COO)_2_·2H_2_O] (Fluka AG, Buchs, Switzerland) solution (dissolution of 2.19 g of salt and 3 mL of acetic acid in water with final volume of 100 mL) respectively were added [18]. The volume was filled with HPLC grade water (Lab-Scan Analytical Sciences) at 100 mL. Samples were thoroughly stirred, left for 10 min, filtered, and used for sugar content analysis with HPLC.

#### 2.9.2. HPLC Analysis

The sucrose, glucose, and fructose content of dough samples was determined with HPLC (HP 1100—Hewlett Packard, Waldbronn, Germany) equipped with a refractive index detector (HP 1047Α). A Nucleosil Carbohydrate EC 250/4 (Macherey-Nagel, Duren, Germany) column was used (flow rate 1.5 mL/min, injection volume 20 μL). The mobile phase was acetonitrile/water (HPLC grade) at the ratio of 80/20 [19]. Extra pure d(+)-Sucrose, d(−)-Fructose (Riedel-de Haen AG, Seelze, Germany) and d(+)-Glucose anhydrous (Panreac Quimica SA, Castellar del Vallès, Spain) were used as reference materials.

All the above measurements/analyses were performed in triplicate.

### 2.10. Statistical Analysis

Statistical elaboration of the data (ANOVA and Duncan’s test for significant differences) and principal component analysis (PCA) were performed using Statistica 8.0 (StatSoft, Tulsa, OK, USA).

## 3. Results and Discussion

Certain characteristics are determinant for the quality of bakery products and have commonly been examined by several researchers [20,21,22]. In this work, weight loss, specific volume, crust and crumb color, as well as the textural and sensorial characteristics of the samples were assessed in order to evaluate the impact of the addition of vital wheat gluten in frozen dough products.

### 3.1. Weight Loss

**Figure 1:**
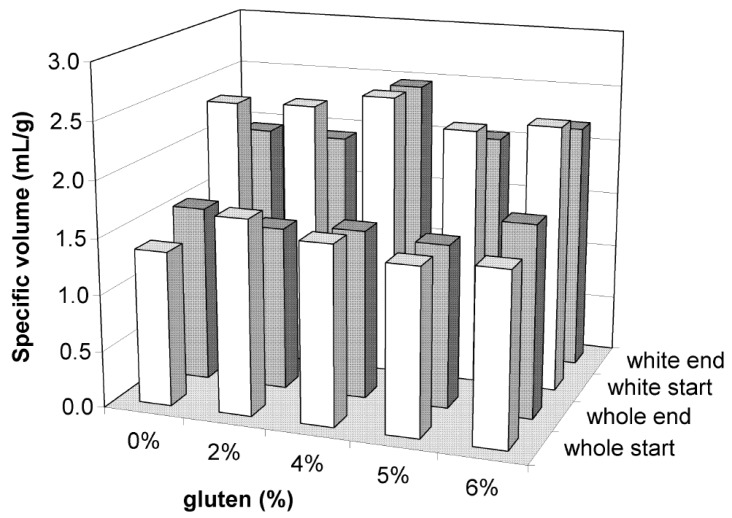
Weight loss (%) during baking of bread samples made from white (start = 11 days, end = 111 days) and whole wheat flour (start = 10 days, end = 110 days).

Weight loss during baking of the samples, should be monitored as it is considered important for the production of bakery products with standard mean weight and sufficient yield. The relative weight loss of all samples from white flour varied between 10.2 and 12.5% while for those from whole-wheat flour between 9.5% and 10.9% (Figure 1). Results indicate that the addition of gluten in most cases caused a decrease in weight loss compared to control samples (*p* < 0.05) which was more intense in samples supplemented with gluten at the level of 5% and 6%. This can be attributed to the increased water holding capacity of samples with added vital wheat gluten. The lowest weight loss fluctuations during frozen storage were observed in samples from white flour with 6% gluten and in samples from whole-wheat flour with 5% gluten.

### 3.2. Specific Volume

**Figure 2:**
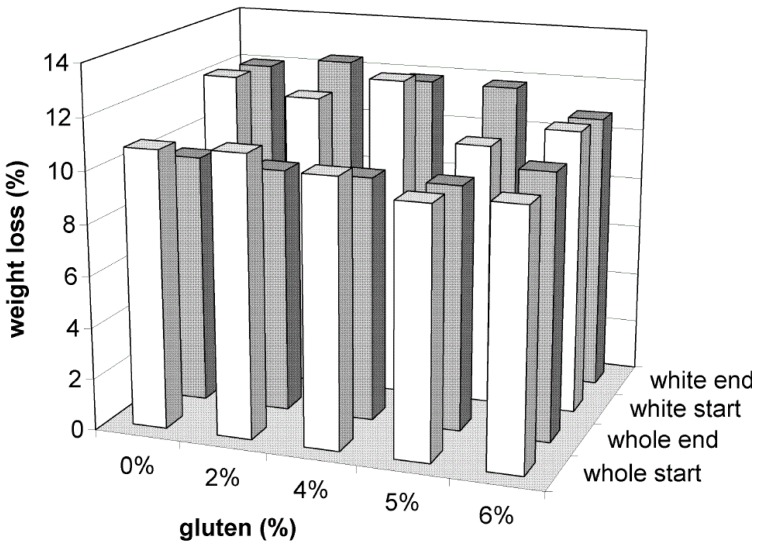
Specific volume (mL/g) of bread samples made from white (start = 11 days, end = 111 days) and whole wheat flour (start = 10 days, end = 110 days).

Specific volume measurements are indicative of dough inflating ability and ovenspring. The specific volume of all samples from white flour varied between 1.9 and 2.5 mL/g while for those from whole-wheat flour between 1.4 and 1.7 mL/g (Figure 2). The addition of gluten in almost every case caused an increase in the specific volume of samples (*p* < 0.05). This was probably due to the viscoelastic properties of gluten which increase the gas holding ability of dough [20]. From the experimental results, it was shown that for white flour samples a 4% addition of gluten could provide a sufficient bread expansion while for whole-wheat flour samples a 6% addition was required respectively.

### 3.3. Color Measurement

Crust and crumb color measurement in bread samples is associated with their appearance and sensorial attraction. Crust color varied between 23 and 34.3 and crumb color between 16.3 and 18.4 for samples from white flour while in those from whole-wheat flour between 22.2–25.6 and 21–22.4, respectively (data not shown). The addition of vital wheat gluten affected significantly (*p* < 0.05) only the crust color of samples from white flour causing an increase which, apart from samples with 5% gluten, was proportional to the percentage of addition. The most stable samples during frozen storage appeared to be those with 4% and 6% gluten in samples from white flour and with 4% gluten in samples from whole-wheat flour. Crumb color was not seriously induced in both cases.

### 3.4. Texture Analysis

Texture analysis, in both dough and bread samples, is very important as it is correlated with formulation and processing and can provide useful information about samples’ quality characteristics [1]. The addition of vital wheat gluten increased dough firmness (*p* < 0.05) both in samples from white flour and whole-wheat flour. This was expected, due to the increased solid/water ratio as well as the increase of water binding capacity of the dough with added gluten, and was also verified by the results of the Analysis of Variance (ANOVA). In the case of white flour, control samples were significantly different from samples supplemented with gluten while in the case of whole-wheat flour, only samples with 5% and 6% gluten were significantly different. During storage, as expected due to reduced CO_2_ production, there was a slight increase in dough firmness (from 0.77 to 1.026 N for control samples, 1.011 to 1.262 N for 2% gluten, 1.21 to 1.381 N for 4% gluten, 0.961 to 1.041 N for 5% gluten, and 0.885 to 0.991 N for 6% gluten) which however was not considered statistically significant in samples from white flour. Dough firmness in samples from whole-wheat flour increased significantly after 79 days of frozen storage.

As far as crust firmness is concerned, measurements indicated that the addition of vital gluten did not show a significant effect both in samples made from white and whole-wheat flour. However a slight trend of increase was observed at the highest levels of addition. Storage time, on the contrary, had a significant influence on crust firmness leading to higher firmness values at prolonged frozen storage. These findings are in agreement with others similar studies, e.g., Phimolsiripol, *et al.* [23] found that as frozen storage time increased, the firmness of frozen dough bread increased as well, and the bread tended to have a coarser texture. Average values (during storage) of dough and crust firmness are presented in Table 1. Concerning crumb firmness, there were no significant differences between samples with different levels of vital wheat gluten. Only a slight increase of firmness can be reported in all samples during frozen storage.

**Table 1:** Average values (during storage of 111 days for white flour samples and 110 days for whole flour samples) of dough and crust firmness.

Gluten Level	Dough Firmness (N)		Crust Firmness (N)	
	White Flour	Whole-Wheat Flour	White Flour	Whole-Wheat Flour
0%	0.755 ^a^*	2.313 ^a^	13.951 ^a^	57.661 ^a^
2%	1.038 ^b^	2.532 ^a,b^	12.761 ^a,b^	57.060 ^a^
4%	1.039 ^b^	2.573 ^ab^	11.041 ^b^	60.253 ^a^
5%	0.990 ^b^	3.488 ^c^	14.266 ^a^	71.464 ^b^
6%	1.063 ^b^	3.011 ^b^	14.210 ^a^	63.490 ^a^

* Values marked with different letters in each column are significantly different (*p* < 0.05).

### 3.5. Sensory Evaluation

**Figure 3:**
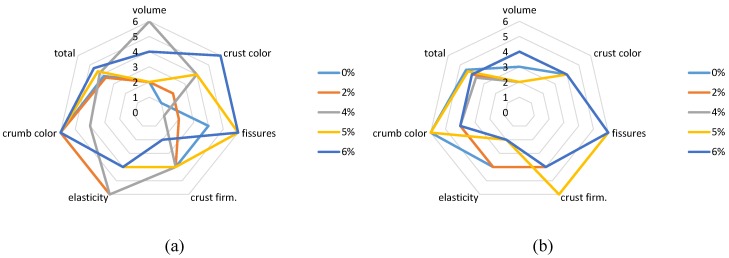
Sensory characteristics of bread samples made from white flour (**a**) and whole-wheat flour (**b**) after frozen storage for 111 and 110 days, respectively.

Results after prolonged storage (111 days for white flour samples and 110 days for whole-wheat flour samples respectively) indicate that in the first case, the best scores were observed for samples with 4% (highest volume) and 6% gluten (better crust color). In the second case, the highest scores for volume were observed in samples with 6% gluten and for crust firmness in 5% gluten (Figure 3a,b). In most of the cases, sensory evaluation data coincide with the findings from analytical measurements. Total score was calculated as the average of all measured features (bread volume and fissures, crust color and firmness, crumb elasticity and color).

### 3.6. Principal Component Analysis

**Figure 4:**
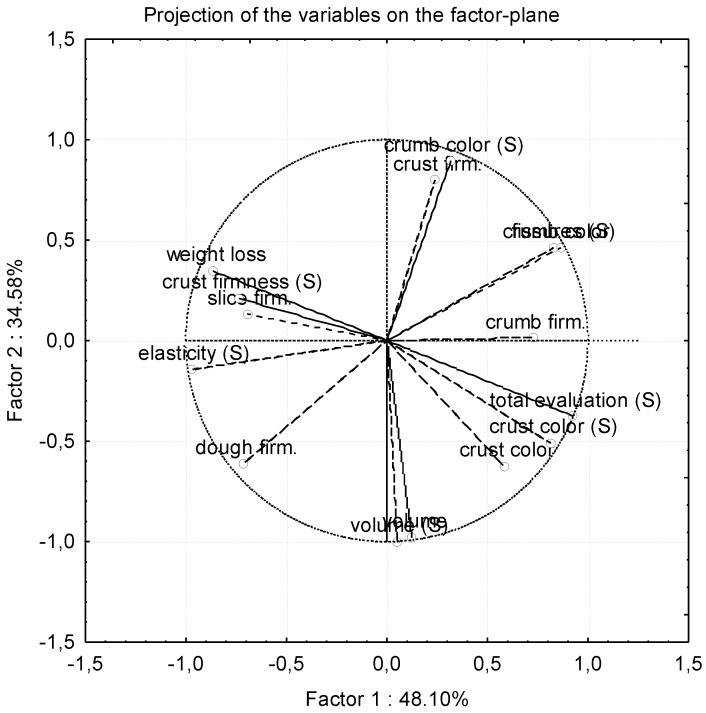
Principal Component Analysis (PCA) variables plot (firm. refers to firmness).

In order to study the overall influence of the above parameters in samples’ behavior, principal component analysis (PCA) was performed. PCA is a useful data reduction technique which transforms the original variables to a smaller number of uncorrelated variables. This facilitates the interpretation of the data and the detection of its internal structure according to variance. The number of factors was reduced to 2 which explains 83% of the total variance of the data. The occurring plots for the variables tested are presented in Figure 4.

Results indicate that samples’ objective and sensorial expansion are strongly correlated with each other. This also applies for crust color. Total sensorial evaluation is more closely correlated to crust color, and weight loss to crust firmness (from sensory evaluation) and slice firmness respectively. Furthermore, weight loss is negatively correlated to crust color and total sensorial evaluation, elasticity to crumb firmness, and dough firmness to fissures created during baking and oven expansion, respectively.

From the cases plot (not shown), it is observed that samples made from different flour types are distinctly grouped, indicating significant differences in their behavior. However, there is no obvious segregation between samples at different storage intervals.

#### DSC and HPLC Studies

In order to further investigate the role of vital wheat gluten in frozen bakery products additional studies were performed in selected samples. More specifically, DSC and HPLC analysis (sugar content) was conducted in control samples from white flour as well as samples containing 6% of vital wheat gluten.

### 3.7. DSC Analysis

**Figure 5:**
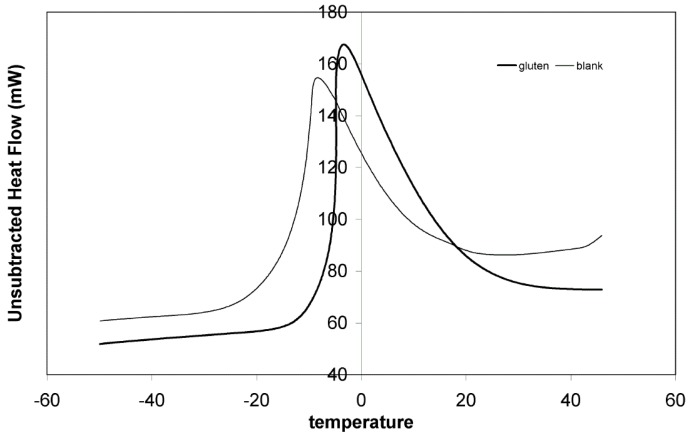
Differential Scanning Calorimetry (DSC) diagrams comparison between samples made from white flour supplemented with 6% gluten and control samples.

Phase and state transitions, which occur at sub-zero temperatures, are associated with ice-recrystallization rates and diffusion-controlled reactions and may affect products’ quality characteristics [24,25]. Differential thermal analysis using DSC can provide useful information, which, in the case of dough freezing, are associated with storage stability and process/formula optimization [19]. The melting peak of ice for samples made from white flour supplemented with vital wheat gluten at the level of 6% was found to have its onset at −12 °C while for control samples at −23 °C (Figure 5). Although the addition of proteins in a food system should depress its freezing point, it was found in this study that gluten enriched samples froze more rapidly diminishing the quality loss occurring during the freezing phase. This can be attributed to the change in freezable water content due to the high water absorption of gluten. As in the study of Laaksonen & Roos [19], who studied the physical state of wheat dough at sub-zero temperatures using DSC, Dynamic-mechanical Analysis (DMA), and Dielectric Analysis (DEA), no other transitions were noticed during DSC analysis.

### 3.8. Low Molecular Sugar Content

**Figure 6:**
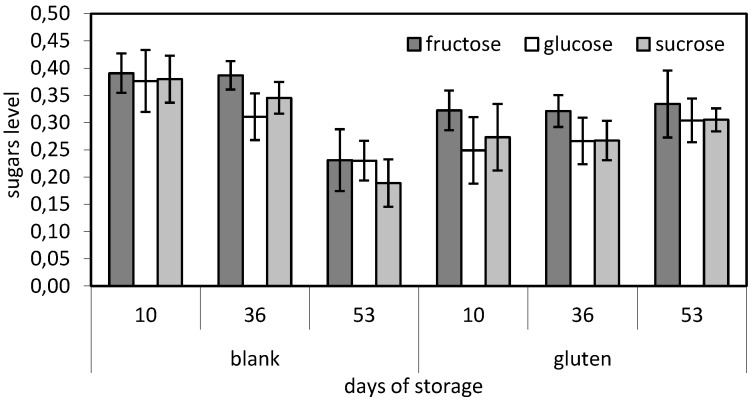
Low molecular sugar (fructose, glucose, sucrose) content comparison between samples made from white flour supplemented with 6% gluten and control samples.

The determination of the soluble carbohydrates remaining after dough fermentation can be important since they participate in browning reactions during baking, contributing to the sensory characteristics of bread, and are believed to enhance bread freshness and texture [26]. In the current study, it was observed that control samples allocated higher sugar content during the first days of frozen storage and presented a fast degrading behavior afterwards (Figure 6). On the contrary, samples supplemented with 6% gluten presented a sufficiently stable behavior during storage (*p* < 0.05). More specifically sucrose content remained constant during frozen storage while fructose and glucose presented a slight increase. These findings reinforce the fact that the addition of gluten helps to maintain dough stability during freezing which, by extension, can affect yeast cells’ survival.

## 4. Conclusions

Freezing and storage of dough at sub-zero temperatures is known to impair dough strength and, inevitably, bread’s quality characteristics. Findings from this study indicate that the addition of vital wheat gluten at a level above 2% can significantly improve parameters such as loaf volume, color, texture, and thermal behavior of the samples. Dough supplementation with gluten led to variable breadmaking potential which altered according to the addition level and the type of flour used for the preparation of samples. More specifically, in the case of white flour the most stable samples were those containing 4% and 6% gluten while in the case of whole-wheat flour those with 4% and 5% gluten.

Thermal analysis with DSC showed that the addition of gluten raises the freezing point of dough samples reducing quality loss due to thermal shock. Finally measurements from low molecular sugar content analysis using HPLC indicated that the supplementation of dough with gluten can significantly increase its stability and improve its strength.

During storage the most prominent changes were observed in samples’ textural characteristics. More specifically, dough firmness was slightly increased in samples from white flour, while in samples from whole-wheat flour, it was increased significantly after 79 days of frozen storage. Crust firmness was also increased noticeably after prolonged frozen storage.

However, in order to confirm the findings from the current work, research should be continued with different storage conditions (different freezing rates and storage temperatures). Also, the quality characteristics of gluten/flour proteins should be further examined as it is found that dough strength is not only designated by protein content but mainly by their quality.

## References

[1] Giannou V., Tzia C. (2007). Frozen dough bread: Quality and textural behavior during prolonged storage—Prediction of final product characteristics. J. Food Eng..

[2] Baier-Schenk A., Handschin S., Von Schonau M., Bittermann A.G., Bachi T., Conde-Petit B. (2005). *In situ* observation of the freezing process in wheat dough by confocal laser scanning microscopy (CLSM): Formation of ice and changes in the gluten network. J. Cereal Sci..

[3] Selomulyo V.O., Zhou W. (2007). Frozen bread dough: Effects of freezing storage and dough improvers. J. Cereal Sci..

[4] Cauvain S. (2015). Technology of Breadmaking.

[5] Ribotta P.D., Leοn A.E., Anon M.C. (2001). Effect of freezing and frozen storage of doughs on bread quality. J. Agric. Food Chem..

[6] Giannou V., Tzia C., LeBail A., Sun D.-W. (2005). Quality and safety of frozen bakery products. Handbook of Frozen Food Processing and Packaging.

[7] Zhou W., Hui Y.H. (2014). Bakery Products: Science and Technology.

[8] Borla O.P., Motta E.L., Saiz A.I., Fritz R. (2004). Quality parameters and baking performance of commercial gluten flours. Food Sci. Technol.—LEB.

[9] Day L., Augustin M.A., Batey I.L., Wrigley C.W. (2006). Wheat-gluten uses and industry needs. Trends Food Sci. Technol..

[10] Figoni P. (2008). How Baking Works: Exploring the Fundamentals of Baking Science.

[11] Cauvain S., Young L., Cauvain S., Young L. (2001). Flours. Baking Problems Solved.

[12] Cauvain S.P., Cauvain S.P. (2003). Breadmaking—An overview. Bread Making, Improving Quality.

[13] Goesaert H., Brijs K., Veraverbeke W.S., Courtin C.M., Gebruers K., Delcour J.A. (2005). Wheat flour constituents: How they impact bread quality, and how to impact their functionality. Trends Food Sci. Technol..

[14] Gallagher E., Gormley T.R., Arendt E.K. (2004). Recent advances in the formulation of gluten-free cereal-based products. Trends Food Sci. Technol..

[15] Koh B.K., Lee G.C., Lim S.T. (2005). Effect of amino acids and peptides on mixing and frozen dough properties of wheat flour. J. Food Sci..

[16] AACC (2000). Method 10-05.01: Guidelines for Measurement of Volume by Rapeseed Displacement.

[17] MacDougall D.B., MacDougall D.B. (2002). Colour measurement of food, Principles and practice. Colour in Food, Improving Quality.

[18] Egan H., Kirk R.S., Sawyer R. (1981). Pearson’s Chemical Analysis of Foods.

[19] Laaksonen T.J., Roos Y.H. (2000). Thermal, dynamic-mechanical, and dielectric analysis of phase and state transitions of frozen wheat doughs. J. Cereal Sci..

[20] Dobraszczyk B.J., Morgenstern M.P. (2003). Rheology and the breadmaking process (Review). J. Cereal Sci..

[21] Hung P.V., Morita N. (2004). Dough properties and bread quality of flours supplemented with cross-linked cornstarches. Food Res. Int..

[22] Azizi M.H., Rao G.V. (2005). Effect of storage of surfactant gels on the bread making quality of wheat flour. Food Chem..

[23] Phimolsiripol Y., Siripatrawan U., Tulyathan V., Cleland D.J. (2008). Effects of freezing and temperature fluctuations during frozen storage on frozen dough and bread quality. J. Food Eng..

[24] Rasanen J., Blanshard J.M.V., Mitchell J.R., Derbyshire W., Autio K. (1998). Properties of frozen wheat doughs at subzero temperatures. J. Cereal Sci..

[25] Bot A. (2002). Differential scanning calorimetric study on the effects of frozen storage on gluten and dough. Cereal Chem..

[26] Lefebvre D., Gabriel V., Vayssier Y., Fontagne-Faucher C. (2002). Simultaneous HPLC determination of sugars, organic acids and ethanol in sourdough process. Food Sci. Technol.—LEB.

